# Medical costs and quality-adjusted life years associated with smoking: a systematic review

**DOI:** 10.1186/s12889-016-3319-z

**Published:** 2016-07-27

**Authors:** Shari P. Feirman, Allison M. Glasser, Lyubov Teplitskaya, David R. Holtgrave, David B. Abrams, Raymond S. Niaura, Andrea C. Villanti

**Affiliations:** 1 The Schroeder Institute for Tobacco Research and Policy Studies at Truth Initiative, 900 G Street NW, Fourth Floor, Washington, DC 20001 USA; 2 Evaluation Science and Research, Truth Initiative, 900 G Street NW, Fourth Floor, Washington, DC 20001 USA; 3 Zanvyl Krieger School of Arts and Sciences, Johns Hopkins University, 3400 N. Charles Street, Baltimore, MD 21218 USA; 4 Department of Health, Behavior and Society, Johns Hopkins Bloomberg School of Public Health, 615 N. Wolfe Street, Baltimore, MD 21205 USA; 5 Georgetown University Medical Center, Lombardi Comprehensive Cancer Center, 3970 Reservoir Road NW E501, Washington, DC 20007 USA

**Keywords:** Smoking, QALY, Medical costs

## Abstract

**Background:**

Estimated medical costs (“T”) and QALYs (“Q”) associated with smoking are frequently used in cost-utility analyses of tobacco control interventions. The goal of this study was to understand how researchers have addressed the methodological challenges involved in estimating these parameters.

**Methods:**

Data were collected as part of a systematic review of tobacco modeling studies. We searched five electronic databases on July 1, 2013 with no date restrictions and synthesized studies qualitatively. Studies were eligible for the current analysis if they were U.S.-based, provided an estimate for Q, and used a societal perspective and lifetime analytic horizon to estimate T. We identified common methods and frequently cited sources used to obtain these estimates.

**Results:**

Across all 18 studies included in this review, 50 % cited a 1992 source to estimate the medical costs associated with smoking and 56 % cited a 1996 study to derive the estimate for QALYs saved by quitting or preventing smoking. Approaches for estimating T varied dramatically among the studies included in this review. T was valued as a positive number, negative number and $0; five studies did not include estimates for T in their analyses. The most commonly cited source for Q based its estimate on the Health Utilities Index (HUI). Several papers also cited sources that based their estimates for Q on the Quality of Well-Being Scale and the EuroQol five dimensions questionnaire (EQ-5D).

**Conclusions:**

Current estimates of the lifetime medical care costs and the QALYs associated with smoking are dated and do not reflect the latest evidence on the health effects of smoking, nor the current costs and benefits of smoking cessation and prevention. Given these limitations, we recommend that researchers conducting economic evaluations of tobacco control interventions perform extensive sensitivity analyses around these parameter estimates.

**Electronic supplementary material:**

The online version of this article (doi:10.1186/s12889-016-3319-z) contains supplementary material, which is available to authorized users.

## Background

Decision-makers, faced with limited financial resources, must typically consider the cost and cost-effectiveness of different options when deciding which policies and programs to implement [1]. As recommended by the Panel on Cost-Effectiveness in Health and Medicine for economic evaluations [1–3], cost-utility analyses typically express outcomes in terms of cost per quality-adjusted life year (QALY), a standard measure that allows decision-makers to make comparisons across different types of interventions.

Modeling the potential impacts of policies on population-level health is of particular interest to the field of tobacco control given the current regulatory environment in the United States (U.S.). The Food and Drug Administration (FDA) is required to evaluate the economic impact of proposed regulatory options [4] and has expressed interest in employing mathematical modeling methods to assess the effects of potential policies [5, 6]. The lifetime medical costs associated with smoking (“T”) and the number of quality-adjusted life years associated with smoking prevention or cessation (“Q”) are essential drivers of the cost-effectiveness of a policy option, but methodologically challenging to estimate for two reasons: first, the true values of these parameters can change with evolving evidence on the harms of smoking [7–9] and rising medical costs; and second, the costs and benefits of smoking prevention and cessation are distal and do not accrue until years following an intervention.

The current study builds upon existing reviews of economic evaluations in tobacco control [8, 10]. While these previous reviews focused on synthesizing the findings of economic evaluations [8, 10] and on standardizing cost-effectiveness ratios to facilitate comparisons between interventions [8], they do not provide in-depth assessments of the models used to generate findings for individual studies. The aim of the current study is to address this gap by providing a detailed investigation into how the parameters T and Q have been estimated in tobacco control literature.

## Methods

Data for this study were collected as part of a systematic review of studies that employed mathematical modeling methods to project tobacco-related outcomes [11]. The methods and overarching descriptive findings from that review can be found elsewhere [11, 12], and PRISMA guidelines have been adhered to. Briefly, we searched five electronic databases (CINAHL, Embase, PsychINFO, PubMed, and EconLit) on July 1, 2013 with no date restrictions and synthesized studies qualitatively (Additional file 1: Table S1). Only peer-reviewed, published literature in English language was eligible for inclusion. Models that project only retrospectively (i.e., analyze the historical burden of disease) were excluded from this review. Studies that model individual smoker trajectories that do not also project population-level outcomes were also excluded. Animal studies, human genetics studies, and posters and abstracts without full text records were not included this review.

### Eligible studies and search strategy

In the current analysis, studies were eligible if they estimated an economic outcome [11]; were conducted in the U.S.; provided an estimate for Q; and used a societal perspective and lifetime analytic horizon to estimate T. We included only U.S.-based studies because the U.S. healthcare system differs from those of most other industrialized countries and, thus, costs spent to treat a disease in the U.S. are not necessarily comparable to those spent on the same condition elsewhere. We included only studies that used a societal perspective and lifetime analytic horizon to estimate T because studies without these specifications could reasonably exclude T from their analyses; we wanted to capture all studies that were structured in such a way that, from a methodological perspective, should have provided an estimate for T. Two pairs of coders independently reviewed the title and abstract of each included record, then two coders reviewed the full texts of articles that met the inclusion criteria and exhibited moderate agreement (k = 0.53) during this phase of the review process.

### Data extraction

Three authors conducted data extraction for each study in pairs (SF, AG, LT). We employed a data extraction form with open-ended questions to capture the heterogeneous ways in which authors describe their methods for calculating Q and T. The form included items about the target population for which Q was estimated, methods for estimating T and Q (including cited sources), discounting practices, and discussion around decisions not to estimate T.

### Analysis

Given the goals of this review and the heterogeneity of the included studies, we synthesized studies qualitatively. We performed our analysis in two stages. First, we identified the methods used to estimate Q and T in each included study. Second, we identified sources that were cited for estimating these parameters and investigated these primary sources. While we did not conduct a formal risk of bias assessment for the studies included in this review, we evaluated the quality and relevance of these frequently cited sources, based on criteria developed to address this review’s questions, to better understand how researchers are estimating Q and T.

We developed tables to describe the methods used to estimate both parameters. We included information about whether the authors discounted these parameters at 3 %, which is standard practice [3]. We also documented whether each study assessed uncertainty with regard to estimating these parameters. We tagged studies as having explored uncertainty for these parameter estimates if they either ran their analysis with multiple discount rates for the same base value or employed multiple base values.

To visualize the network of citations used to estimate T and Q and illustrate the chronological link between studies, we conducted a bibliometric analysis using HistCite Version 2009.08.24. We examined the studies included in this review and the studies they cited as sources for estimating T and Q. The analysis was limited to studies indexed in Web of Science.

## Results and discussion

**Fig. 1:**
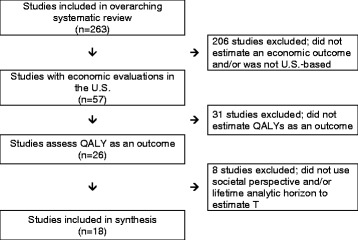
Flowchart of included studies

Fifty-seven studies from our overarching systematic review estimated economic outcomes [11] and were conducted in the U.S. Of those, 26 studies estimated QALYs as an outcome [13–38]. Eighteen of those studies employed a societal perspective and lifetime analytic horizon to estimate T and, thus, were included in our review. Years of publication ranged from 1997 to 2012. The majority of studies targeted the general population, and evaluated smoking cessation programs or policies. The study selection process is illustrated in Fig. 1.

### Medical costs attributable to smoking (T)

Information on estimates of T can be found in Table 1. Five studies did not estimate T in their analyses [18, 23, 24, 26, 36]. Of the 13 studies that provided estimates for T, 11 studies (85 %) discounted T by 3 % [13, 14, 21, 25, 27, 29, 31, 34, 35, 37, 38]. Nine studies (69 %) accounted for uncertainty in their estimate by using different assumptions for the value of T [21, 25, 27, 29, 31, 34, 35, 37, 38].

**Table 1:** Methods for estimating medical costs attributable to smoking (T)

First author, year published	Source for lifetime medical costs attributable to smoking	Discounted at 3 %	Assessment of uncertainty
Ahmad, 2005 [13]	Hodgson, 1992 [39]	Yes	No
	^a^Medical Expenditure Panel Survey [66]		
Ahmad, 2005 [14]	Hodgson, 1992 [39]	Yes	No
	^a^Medical Expenditure Panel Survey [66]		
Ahmad, 2005 [15]	Hodgson, 1992 [39]	No	No
	Medical Expenditure Panel Survey [66]		
Ahmad, 2008 [16]	Hodgson, 1992 [39]	Not stated	No
	^a^Medical Expenditure Panel Survey [66]		
Cromwell, 1997 [18]	Did not include lifetime medical costs associated with smoking; included discussion	N/A	N/A
Holtgrave, 2009 [21]	Wang, 2001 [38]	Yes	Yes
	Sloan, 2004 [45]		
	Warner, 2004 [43]		
Javitz, 2004 [23]	Did not include lifetime medical costs associated with smoking; included discussion	N/A	N/A
Javitz, 2011 [24]	Did not include lifetime medical costs associated with smoking; included discussion	N/A	N/A
Kahn, 2008 [25]	Kaiser Permanente Southern California (no citation given)	Yes	Yes
	The Diabetes Prevention Program Research Group [67]		
Keeler, 2002 [26]	Did not include lifetime medical costs associated smoking; did not include discussion	N/A	N/A
Knight, 2010 [27]	Halpern, 2003 [68]	Yes	Yes
	Kutikova, 2005 [69]		
	Tsevat, 2001 [70]		
	Taylor, 1996 [71]		
	Stanford, 1999 [72]		
McMahon, 2011 [29]	Cipriano, 2010 [73]	Yes	Yes
	SEER-Medicare linked data (no citation given)		
Ruger, 2008 [31]	Gold, 1996 [41]	^b^Yes	Yes
	Cromwell, 1997 [18]		
	Fiscella, 1996 [20]		
	Rogers, 1991 [40]		
	Miller, 2001 [74]		
Solberg, 2006 [34]	Musich, 2003 [75]	Yes	Yes
	Keehan, 2004 [76]		
	Hoffman, 2001 [77]		
Tengs, 2001 [35]	Hodgson, 1992 [39]	Yes	Yes
Tran, 2002 [36]	Did not include lifetime medical costs associated smoking; did not include discussion	N/A	N/A
Villanti, 2012 [37]	Hodgson, 1992 [39]	Yes	Yes
	Warner, 2004 [43]		
	Cromwell, 1997 [18]		
Wang, 2001 [38]	Hodgson, 1992 [39]	Yes	Yes
	Manning, 1989 [44]		

^a^No citation was given; “Medical Expenditure Panel Survey” was only referenced in the text

^b^States that medical costs were discounted but does not specify at 3 %. We assume a 3 % discount rate was used because the paper appears to reference the U.S. Panel on Cost-Effectiveness in Health and Medicine guidelines for performing the analysis

### Summary of medical costs attributable to smoking (T)

#### Hodgson

Seven papers (54 %) directly cited a 1992 paper by Hodgson [39] as a source for estimating T [13–16, 35, 37, 38]. A study conducted by Holtgrave et al. [21] cited Wang et al. [38] as a source for estimating T. Wang et al., in turn, cited Hodgson’s paper. Thus, Holtgrave et al. indirectly cited Hodgson for this estimate. A study by Ruger et al. [31] also seems to indirectly cite Hodgson. Ruger et al. cited three studies [18, 20, 40] as sources for their upper value estimate for T. Only one of these studies, conducted by Cromwell et al. [18], appears to include a discussion of the value used by Ruger et al. Cromwell et al.’s study did not include a primary calculation for this value; it cited a publication by Gold et al. [41] We could not find this value in the publication by Gold et al.; this value appears to be the estimate of the excess lifetime medical expenditures incurred by smokers presented in Hodgson’s paper.

Four of the seven (57 %) studies that directly cited Hodgson’s estimate did so only to estimate T for adults; they employed other methods to estimate T for youth [13–16]. In these four studies, no sensitivity analysis was conducted around the value of T for adults. Villanti et al. and Wang et al. [37, 38] used Hodgson’s estimate as the highest value in a sensitivity analysis, while Holtgrave et al. [21] used Hodgson’s estimate as the base-case estimate.

Hodgson estimated the medical expenditures associated with smoking based on gender, age, smoking intensity and survival status. He considered ages from 17 years until death (17–34, 35–44, 45–54, 55–64, 65–74, 75–84, 85+) and four levels of smoking intensity. The analysis incorporated expenditures from Medicare, Medicaid, direct costs and other private costs (primarily from private insurance). Hodgson based his estimates on data from the National Health Interview Survey (NHIS) (hospital and physician services); National Nursing Home Survey and National Health and Nutrition Examination Survey Epidemiologic Follow-up Study (nursing-home expenditures); American Cancer Society’s Cancer Prevention Study II (mortality); and National Medical Care Utilization and Expenditure Survey and Medicare data files (medical care charges). Hodgson does not specify the years during which these surveys were fielded. He employed a 3 % discount rate. Additional analyses were conducted with a 5 % discount rate.

### Medical Expenditure Panel Survey

Four of the seven (57 %) studies citing Hodgson also cited the Medical Expenditure Panel Survey (MEPS) [42] as a source for estimating T [13–16]. These studies used MEPS data to estimate T for youth and Hodgson’s data to estimate T for adults. The MEPS is composed of two major components; one collects data from individual households and their medical providers, and one collects data on employer-based health insurance. The MEPS also surveys healthcare organizations and facilities identified by respondents [42]. None of the authors who cited the MEPS as a source for estimating T specifically described which MEPS data they used for their analyses. No sensitivity analyses were conducted around this value.

#### Warner et al.

Two studies [37, 43] (15 %) employed an estimate generated by Warner et al. [43] for their lowest value in a sensitivity analysis for T. To obtain this estimate, Warner et al. used data from existing literature and managed care organizations (MCOs) to inform a computer simulation model that tracks members of a hypothetical MCO. Warner et al. value T negatively; the estimate assumes that former smokers incur greater medical costs compared to continuing smokers as a result of a longer lifespan. The authors discounted costs at 3 %.

#### Manning et al.

Wang et al. [38] used an estimate by Manning et al. [44] as their lowest estimate for T. Holtgrave et al. [21], who indirectly cited Manning et al. (via Wang et al.) for this estimate, used this value in their base-case analysis. The sources of this estimate were the RAND National Health Insurance Experiment and the NHIS. In calculating the differential medical care costs for smokers versus never smokers, Manning et al. controlled for risky behaviors such as alcohol use. Thus, Manning et al.’s estimate of T is lower than Hodgson’s.

#### Sloan et al.

Holtgrave et al. used Sloan et al.’s [45] estimate to calculate their highest estimate of T in a sensitivity analysis. Sloan et al. estimated the annual private medical care cost of smoking for a 24 year-old. Holtgrave et al. calculated lifetime medical costs by assuming that this cost would be incurred annually over many years; they discounted their estimate at 3 % over 27 years.

### Other methods

Four studies [25, 27, 29, 34] (31 %) estimated T by using other sources that directly tracked medical costs incurred and/or by consulting literature on the costs of treating specific tobacco-related diseases. These sources can be found in Table 1. Two of these studies did not provide details about their sources, such as values of the inputs in their models [25, 27].

#### Studies that omitted T

Five studies (38 %) excluded estimates of T from their analyses [18, 23, 24, 26, 36]. Two of these studies did not explicitly note this omission [26, 36]. One source acknowledged the omission without discussion [24]. Two studies [18, 23] cited difficulty determining whether smoking cessation produces long-term increased or decreased healthcare costs in discussing their omission of T.

#### Studies that valued T at $0

Two studies (15 %) included an estimate of $0 for T as their base-case estimates [31, 37]. In discussing their rationale for valuing T at $0, the authors of these studies cited previously published studies [18, 20, 23, 41].

#### Synthesis

Hodgson’s estimate, published in 1992, was the most frequently cited source; nine papers cited this study. This estimate, however, was only one of several used across the studies in this review. Estimates of T assumed positive, negative, and $0 values, illustrating the heterogeneity of assumptions used across studies.

### QALYs associated with preventing or quitting smoking (Q)

Information on estimates of Q can be found in Table 2. Most studies estimated Q for adult populations. Two studies focused on youth [21, 38] and five focused on populations that included youth and adults [13–16, 35]. All but two [15, 16] papers discounted QALYs at 3 %. The authors of 10 [18, 21, 23–26, 31, 35–37] of the 18 studies in this review accounted for uncertainty by running their analyses with different values for Q. We identified three preference-based health-state classification systems and one additional method that were used to calculate values and utilities for Q.

**Table 2:** Methods for estimating QALYS associated with quitting or preventing smoking (Q)

First author, year published	Target population	Source for QALY weights	Method of determining QALYS^c^	Discounted at 3 %	Assessment of uncertainty
Ahmad, 2005 [13]	Entire California population	^a^Kaplan, 2007 [49]	QWB Scale	Yes	No
Ahmad, 2005 [14]	Entire U.S. population	^a^Kaplan, 2007 [49]	QWB Scale	Yes	No
Ahmad, 2005 [15]	Entire California population	^a^Kaplan, 2007 [49]	QWB Scale, Other	No	No
		Erikson, 1995 [46]			
Ahmad, 2008 [16]	Entire U.S. population	^a^Kaplan, 2007 [49]	QWB Scale, Other	Not stated	No
		Erikson, 1995 [46]			
Cromwell, 1997 [18]	Adults in the U.S.	Fiscella, 1996 [20]	HUI	Yes	Yes
Holtgrave, 2009 [21]	Youth in the U.S.	Kaplan, 2007 [49]	HUI, QWB Scale	Yes	Yes
		Wang, 2001 [38]			
Javitz, 2004 [23]	Adults in the U.S.	Fiscella, 1996 [20]	HUI, Other	Yes	Yes
		Erickson, 1995 [46]			
Javitz, 2011 [24]	Adults in the U.S.	Fiscella, 1996 [20]	HUI, Other	Yes	Yes
		Erickson, 1995 [46]			
Kahn, 2008 [25]	Adults in the U.S.	Sullivan, 2006 [50]	EQ-5D Index	Yes	Yes
Keeler, 2002 [26]	Adults in the U.S.	Fiscella, 1996 [20]	HUI	Yes	Yes
Knight, 2010 [27]	Adults in the U.S.	^b^Fiscella, 1996^b^	HUI	Yes	^b^Not clear
McMahon, 2011 [29]	Adults in the U.S.	Hanmer, 2006 [51]	EQ-5D Index	Yes	No
Ruger, 2008 [31]	Low-income pregnant women in the U.S.	Fiscella, 1996 [20]	HUI, Other	Yes	Yes
		Cromwell, 1997 [18]			
		Erickson, 1997 [46]			
Solberg, 2006 [34]	Adults in the U.S.	Maciosek, 2005 [78]	Other	Yes	Yes
		Maciosek, 2001 [79]			
		Miscellaneous sources related to stroke [80–86]			
Tengs, 2001 [35]	Entire U.S. population	^a^Kaplan, 2007 [49]	QWB Scale, Other	Yes	Yes
		Erikson, 1995 [46]			
Tran, 2002 [36]	Adults in the U.S.	Fiscella, 1996 [20]	HUI	Yes	Yes
Villanti, 2012 [37]	Adult in the U.S.	Wang, 2001 [38]	HUI	Yes	Yes
		Javitz, 2004 [23]			
Wang, 2001 [38]	Students in the U.S.	Cromwell, 1997 [18]	HUI	Yes	No

^a^Cited as “R. M. Kaplan, 1999, personal communication” in text. For the purpose of this analysis, we assume that the methodology described in [49] was used in this paper

^b^“Utility values for the various model states were also drawn from a variety of literature sources [20–27].” Fiscella was one of these sources. We assume it is for QALYs associated with quitting smoking. Notes that sensitivity analyses were conducted for utility values, but not clear if sensitivity analysis done specifically for utility values associated with quitting smoking

^c^ *HUI* Health Utilities Index, *QWB* quality of well-being scale, *EQ-5D* EuroQol five dimensions questionnaire

#### Health Utilities Index

A paper by Fiscella et al. [20] was the most frequently cited source for obtaining utility values to estimate Q. Seven [18, 23, 24, 26, 27, 31, 36] of the 18 studies (39 %) in this analysis directly cited this paper. Estimates from three additional studies – conducted by Wang et al. [38] Villanti et al. [37] and Holtgrave et al. [21] – were indirectly based on those published by Fiscella et al. [20] Wang et al. [38] cited a paper by Cromwell et al. [18] as their source for estimating Q; Cromwell et al. [18], in turn, based their estimate on the paper by Fiscella et al. [20] Villanti et al. [37] and Holtgrave et al. [21] cited Wang et al. [38] as a source for estimating Q. Villanti et al. [37] also obtained QALY weight estimates from Javitz et al. [23], who based their estimates on those of Fiscella et al. [20].

Fiscella et al. developed age- and gender-specific QALY estimates for smokers and former smokers (age groups: 25–29, 30–34, 35–39, 40–44, 45–49, 50–54, 55–59, 60–64, 65–69). They based their estimates on years of healthy life measures, developed with 1991 NHIS data. Two domains were measured in the NHIS to assess health status: perceived health and role limitation [46]. Fiscella et al. linked the NHIS data to the HUI. The HUI refers to a family of systems that can be used to produce health-related quality of life utility scores [47, 48]. Fiscella et al. noted that the NHIS does not directly ask the questions needed to estimate QALYs from the HUI, but they stated that their estimates were reasonably valid.

#### Quality Of Well-being Scale

Six studies [13–16, 21, 35] (33 %) based their estimates for Q on the Quality of Well-Being (QWB) Scale [49]. In four of these studies, the authors cited only personal communication with RM Kaplan as the source for the estimates [13–15, 35]. For our analysis, we assume that these four studies employed the methodology for estimating utility values for Q described in a 2007 paper by Kaplan et al. [49].

Kaplan et al. estimated age- and gender-specific QALY estimates for individuals aged 18–70. These estimates accounted for smoking intensity. To assess health, the QWB Scale produces preference weights based on 1) symptoms and problems and 2) dysfunction (mobility, physical activity, social activity). Data for the QWB Scale were obtained from pooled 1987, 1990 and 1994 NHIS data. Kaplan et al. noted that the NHIS does not directly ask the questions needed to input data for the QWB Scale, but the authors believe that their method produces good estimates.

#### EQ-5D index

Two studies [25, 29] (11 %) based their QALY estimates on EQ-5D index scores [50, 51]. The EQ-5D provides age- and gender- specific health-related quality of life scores associated with specific medical conditions. To assess quality of life, the EQ-5D measures mobility, self-care, typical activities, pain/discomfort and anxiety/depression [50]. Kahn et al. [25] cited a source that used the EQ-5D index scores based on data from the 2000–2002 MEPS [50]. McMahon et al. [29] cited a source that obtained EQ-5D index scores from the 2001 MEPS [51].

#### Other calculations

Six papers [15, 16, 23, 24, 31, 35] (33 %) cited a study by Erickson et al. [46] as the source for obtaining QALY estimates associated with smoking. Two of these papers attributed authorship of this article to the Centers for Disease Control and Prevention instead of to Erickson et al. [24, 31]. In three studies, Erickson et al.’s data was used to calculate QALYs for youth [15, 16, 35].

Erickson et al. did not specifically present data related to smoking status. Instead, they calculated average health-related quality of life by age (age groups: 0–5, 5–10, 10–15, 15–20, 20–25, 25–30, 30–35, 35–40, 40–45, 45–50, 50–55, 55–60, 65–70, 75–80, 85+) based on 1990 NHIS data. To obtain the values for the QALYs associated with smoking from this data, researchers extrapolated based on other sources of information.

Solberg et al. [34] employed another method whereby they identified QALY weights associated with chronic and acute conditions, and then applied those weights to specific conditions associated with smoking. The authors calculated the weight for stroke separately from other conditions.

#### Synthesis

All of the approaches described are based on surveys of the general population. Two approaches [20, 49] based their QALY estimates on NHIS data that were collected nearly 25 years ago. These studies note that the NHIS does not directly ask the questions needed to estimate QALYs based on the index/scale being used, but that the authors believe their estimates to be valid [20, 49]. The QWB Scale was the only approach to explicitly account for smoking intensity [49]. Erickson et al. was the only source of data used to estimate QALYs for youth [46].

### Visualization of results

**Fig. 2:**
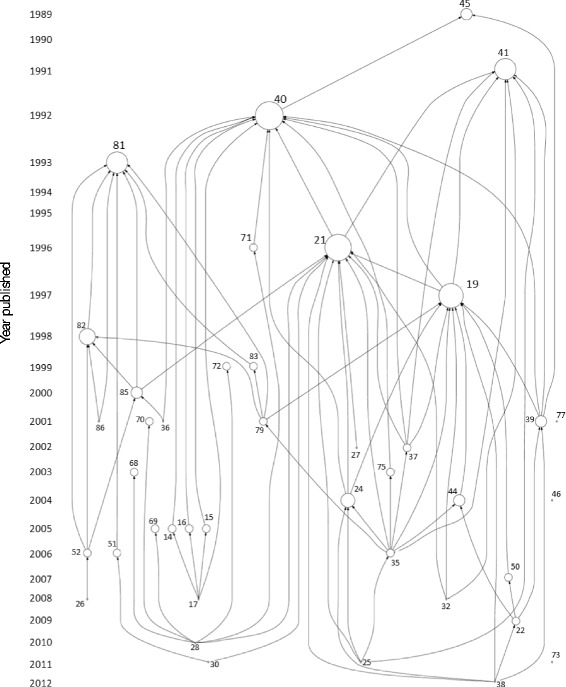
Bibliometric analysis examining the studies included in this review and the papers they cited as sources for estimating T and Q. Numbers next to nodes correspond to citation numbers in the Reference section

The chronological connections between the 18 studies in this review and the studies cited as sources for T and Q can be seen in Fig. 2. Forty-one studies were indexed in Web of Science and are presented in this figure. The largest nodes in this figure represent the publications that were cited most frequently by the studies included in the collection; these values do not represent the number of times a publication is cited in the general literature. The two largest nodes (“40” and “21”) represent the studies published by Hodgson [39] and Fiscella et al. [20]. The top six most frequently cited studies were published in the 1990’s.

## Conclusions

The current study systematically evaluated how researchers have estimated T and Q in economic evaluations of tobacco control interventions. The most frequently cited papers for these estimates were published in 1992 (Hodgson) and 1996 (Fiscella et al.), respectively. These estimates do not take into account the technological advances in the treatment of smoking-related diseases in the past 20 years [52] or up-to-date research about the effects of smoking [9, 53, 54]. Notably, the 2014 Surgeon General’s report concluded that smoking causes more diseases than previously thought [7]. Changes in assumptions about the effects of smoking and the course of illness for individuals with smoking-related diseases may drastically change estimates of T and Q. As a comparison, researchers have published updated estimates of the lifetime medical costs associated with HIV infection as care and treatment for HIV/AIDS has developed, and each update has produced new findings [55, 56].

We noted substantial heterogeneity with regard to the way in which T was valued in the literature. This heterogeneity is problematic because it is difficult to compare studies that employ different methods. Developing a standard approach for estimating T would make it easier to include the true costs of smoking cessation and prevention in comparisons and syntheses of economic evaluations of tobacco control interventions, thus improving the evidence base upon which decisions could be made.

Of the approaches to estimating T, perhaps the most controversial is the assignment of a negative value to the parameter. We argue that treating T in this way is problematic. From a methodological standpoint, estimating T negatively may mean that the researcher has not considered the costs and benefits of smoking cessation equally. A negative value assumes that former smokers incur more medical costs than continuing smokers due to a longer lifespan. While this may be true, it is possible that lifetime earnings resulting from a longer lifespan could outweigh the excess medical costs [57]. From an ethical standpoint, valuing T negatively biases the analysis against an outcome – smoking cessation – that society has deemed to be desirable. Biasing the analysis in this way is inconsistent with a population health approach [58].

Accurately estimating the costs and benefits associated with preventing or quitting smoking has important applications. In 2014, the FDA published a Regulatory Impact Analysis (RIA) to assess the economic impact of a proposed rule [4]. In their analysis, the FDA considered the cost of the proposed regulation to smokers and estimated that the “lost consumer surplus” amounted to a 70 % reduction in the welfare gain experienced by individuals who would quit smoking as a result of the regulation. Prominent economists [59] and others [60] argue that this estimate overvalues the cost and undervalues the benefit of smoking cessation. The development of standard, up-to-date estimates of the costs and benefits associated with smoking cessation and prevention could help avoid the use of estimates in future RIAs that bias the results against the public health goal of saving lives through smoking cessation and prevention. Given the limitations associated with current estimates of T and Q, we recommend that researchers conducting economic evaluations of tobacco control interventions perform extensive sensitivity analyses, including threshold analyses, around these parameter estimates.

We identified additional gaps in the existing literature and suggest directions for future research. First, the most commonly used instruments to classify health states (the HUI, QWB Scale and EQ-5D) employ different methods [61–63], and comparisons of these tools have found that they produce different findings about the health status associated with certain conditions [62, 64]. Future analyses might assess differences in how these instruments estimate the quality of life associated with smoking. Second, authors employing the HUI and QWB Scale to estimate Q noted that the surveys they used estimate health state preferences did not directly ask the questions needed to populate their instruments. Researchers might consider developing a survey that would address this limitation, in addition to exploring other methods to estimate Q. It is unclear whether there is a gold standard method for estimating Q; our findings suggest that the field would benefit from research that investigates and refines such methods. Last, the studies in this review that evaluated prevention-focused interventions [21, 38] employed estimates developed for smoking cessation. To our knowledge, no true estimates for the costs and benefits associated with smoking prevention have been published, even in newer studies examining the impact of smoking prevention [65].

This study considers how T and Q were estimated, and it focuses on U.S. studies. An analysis of how other measures – such as productivity over the lifetime and life-years saved – have been modeled in economic evaluations, and an analysis of international studies, may provide further insight into the current state of economic evaluation research.

## Abbreviations

EQ-5D, EuroQol five dimensions questionnaire; FDA, Food and Drug Administration; HUI, Health Utilities Index; MCOs, managed care organizations; MEPS, Medical Expenditure Panel Survey; NHIS, National Health Interview Survey; Q, number of quality-adjusted life years associated with smoking prevention or cessation; QALY, quality-adjusted life year; QWB, Quality of Well-Being Scale; RIA, Regulatory Impact Analysis; T, lifetime medical costs associated with smoking; U.S., United States

## Additional file

Additional file 1: Table S1. Search Strategy. (DOC 40 kb)
