# Supplementary material for: Medical costs and quality-adjusted life years associated with smoking: a systematic review
Source: BMC Public Health. 2016 Jul 27;16:646. doi: 10.1186/s12889-016-3319-z (PMC4962483; doi:10.1186/s12889-016-3319-z)
Supplement: Additional file 1: Table S1. — Search Strategy. (DOC 40 kb) [file 12889_2016_3319_MOESM1_ESM.doc]

| **Additional file 1 Table S1.** Search Strategy |  |  |
| --- | --- | --- |
| **Concept 1: Modeling** | **Concept 2: Tobacco** | **Restrictions** |
| **PubMed** |  |  |
| "models, theoretical"[majr:noexp] or "models, statistical"[majr:noexp] or “models, economic”[majr] or “computer simulation”[majr:noexp] or "monte carlo method"[mesh] or "decision support techniques"[majr:noexp] or “decision trees”[mesh] or “systems theory”[mesh] or “markov chains”[mesh] or “system dynamics”[tiab] or “agent-based model”[tiab] or “agent-based models”[tiab] or “agent-based modeling”[tiab] or “ agent-based modelling”[tiab] or “simulation model”[tiab] or “decision analysis”[tiab] or “decision framework”[tiab] or “markov”[tiab] or “cost-utility analysis”[tiab] or “cost-utility analyses”[tiab] or “cost-effectiveness analysis”[tiab] or “cost-effectiveness analyses”[tiab] or “cost-benefit analysis”[tiab] or “cost-benefit analyses”[tiab] or “forecasting”[mesh] or “microsimulation”[tiab] or “micro simulation”[tiab] or “monte carlo”[tiab] or "life year"[tiab] or "life years"[tiab] or "smoking-attributable deaths"[tiab] or "smoking attributable deaths"[tiab] or “deterministic”[tiab] or “probabilistic” [tiab] or “stochastic” [tiab] or “dynamic transmission model” [tiab] or “state-transition” [tiab] or “state transition” [tiab] or “discrete event” [tiab] or “continuous event” [tiab] or “analytic horizon” [tiab] or “cohort simulation” [tiab] or “second-order simulation” [tiab] or “first-order simulation” [tiab] or “threshold analysis” [tiab] or “years of healthy life” [tiab] or “decision problem” [tiab] or “transition probabilities” [tiab] or “discount rate” [tiab] | “Smoking”[Mesh] OR "Smoking Cessation"[Mesh] OR “Tobacco Use Cessation Products”[Mesh] OR “Tobacco”[Mesh] OR “Tobacco Products”[Mesh] OR “Tobacco, Smokeless”[Mesh] OR “Smoking”[TI] OR “Tobacco”[TI] OR “Smoker”[TI] or “Smokers”[TI] OR Cigar*[TI] OR “Smokeless”[TIAB] OR E-cigarette*[TIAB] OR Electronic cigarette*[TIAB] OR “Snus”[TIAB] OR “Nicotine”[TIAB]  AND English[lang] | NOT "genetic"[TIAB]  NOT "gene"[TIAB]  Filter: English |
| **Embase** |  |  |
| 'theoretical model'/mj OR 'statistical model'/mj OR 'computer simulation'/mj OR 'disease simulation'/mj OR 'monte carlo method'/mj OR 'decision support system'/mj OR 'decision tree'/mj OR 'systems theory'/mj OR 'economic evaluation'/exp OR 'forecasting'/exp OR 'economic model':ab,ti OR ‘simulation model’:ab,ti OR 'markov':ab,ti OR 'systems dynamics':ab,ti OR 'agent-based model':ab,ti OR 'agent-based models':ab,ti OR 'agent-based modeling':ab,ti OR 'agent-based modelling':ab,ti OR 'decision analysis':ab,ti OR 'decision framework':ab,ti OR 'microsimulation':ab,ti OR 'micro simulation':ab,ti OR ‘life year’:ab,ti OR ‘life years’:ab,ti OR ‘smoking-attributable deaths’:ab,ti OR ‘smoking attributable deaths’:ab,ti OR  ‘deterministic’:ab,ti OR ‘probabilistic’:ab,ti OR ‘stochastic’:ab,ti OR ‘dynamic transmission model’:ab,ti OR ‘state-transition’:ab,ti OR ‘state transition’:ab,ti OR ‘discrete event’:ab,ti OR ‘continuous event’:ab,ti OR ‘analytic horizon’:ab,ti OR ‘cohort simulation’:ab,ti OR ‘second-order simulation’:ab,ti OR ‘first-order simulation’:ab,ti OR ‘threshold analysis’:ab,ti OR ‘years of healthy life’:ab,ti OR ‘decision problem’:ab,ti OR ‘transition probabilities’:ab,ti OR ‘discount rate’:ab,ti | 'smoking'/mj OR 'cigarette smoke'/mj OR 'bidi smoking'/mj OR 'smoking regulation' OR 'smoking cessation'/exp OR 'tobacco'/exp OR 'smokeless tobacco'/exp OR 'electronic cigarette':ab,ti OR 'e-cigarette':ab,ti OR 'snus':ab,ti OR 'nicotine':ab,ti NOT 'cannabis smoking'/exp NOT 'cigarette smoke condensate'/mj | NOT ‘gene’/exp OR ‘genetic’/exp  AND [english]/lim AND [humans]/lim |
| **EconLit** |  |  |
| CC I180 OR CC C530 OR CC J110 OR KW “Simulation” OR CC I120 OR TX “system dynamics” OR “agent-based model” OR “agent-based models” OR “agent-based modeling” OR “ agent-based modelling” OR “simulation model” OR “decision analysis” OR “decision framework” or “markov” OR “cost-utility analysis” OR “cost-utility analyses” OR “cost-effectiveness analysis” OR “cost-effectiveness analyses” OR “cost-benefit analysis” or “cost-benefit analyses” OR “microsimulation” OR “micro simulation” OR “monte carlo” OR "life year" OR "life years" OR “deterministic” OR “probabilistic” OR “stochastic” OR “dynamic transmission model” OR “state-transition” OR “state transition” OR “discrete event” OR “continuous event” OR “analytic horizon” OR “cohort simulation” OR “second-order simulation” OR “first-order simulation” OR “threshold analysis” OR “years of healthy life” OR “decision problem” OR “transition probabilities” OR “discount rate” | KW “Smoking” OR "tobacco" OR TX smokeless OR “Smoking” OR “Tobacco” OR “Smoker” or “Smokers” OR Cigar* OR “Smokeless” OR E-cigarette* OR Electronic cigarette* OR “Snus” OR “Nicotine” OR "smoking-attributable deaths" OR "smoking attributable deaths" | No English Language Limiting Option |
| **CINAHL** |  |  |
| MJ Computer Simulation OR Models, Statistical OR Forecasting OR Cost Benefit Analysis OR Quality-Adjusted Life Years OR TX “system dynamics” OR “agent-based model” OR “agent-based models” OR “agent-based modeling” OR “ agent-based modelling” OR “simulation model” OR “decision analysis” OR “decision framework” or “markov” OR “cost-utility analysis” OR “cost-utility analyses” OR “cost-effectiveness analysis” OR “cost-effectiveness analyses” OR “cost-benefit analysis” or “cost-benefit analyses” OR “microsimulation” OR “micro simulation” OR “monte carlo” OR "life year" OR "life years"  OR “deterministic” OR “probabilistic” OR “stochastic” OR “dynamic transmission model” OR “state-transition” OR “state transition” OR “discrete event” OR “continuous event” OR “analytic horizon” OR “cohort simulation” OR “second-order simulation” OR “first-order simulation” OR “threshold analysis” OR “years of healthy life” OR “decision problem” OR “transition probabilities” OR “discount rate” | MJ Tobacco OR Smoking OR Smoking Cessation OR Smoking – Trends OR Smoking Cessation OR TX smokeless OR “Smoking” OR “Tobacco” OR “Smoker” or “Smokers” OR Cigar* OR “Smokeless” OR E-cigarette* OR Electronic cigarette* OR “Snus” OR “Nicotine” OR "smoking-attributable deaths" OR "smoking attributable deaths" | Only English Language |
| **PsychInfo** |  |  |
| KW cost effectiveness OR economic analysis OR **smoking**-attributable deaths OR quality adjusted **life** expectancy OR economic impact OR SU “Costs and Cost Analysis” OR Health Care Policy OR Simulation OR Decision Making OR Life Expectancy OR TX “system dynamics” OR “agent-based model” OR “agent-based models” OR “agent-based modeling” OR “ agent-based modelling” OR “simulation model” OR “decision analysis” OR “decision framework” or “markov” OR “cost-utility analysis” OR “cost-utility analyses” OR “cost-effectiveness analysis” OR “cost-effectiveness analyses” OR “cost-benefit analysis” or “cost-benefit analyses” OR “microsimulation” OR “micro simulation” OR “monte carlo” OR "life year" OR "life years"  OR “deterministic” OR “probabilistic” OR “stochastic” OR “dynamic transmission model” OR “state-transition” OR “state transition” OR “discrete event” OR “continuous event” OR “analytic horizon” OR “cohort simulation” OR “second-order simulation” OR “first-order simulation” OR “threshold analysis” OR “years of healthy life” OR “decision problem” OR “transition probabilities” OR “discount rate” | KW tobacco control policies OR tobacco control policy OR smoking cessation OR smokeless tobacco OR cession treatment policies OR population smoking prevalence OR tobacco elimination OR cessation programs OR cigarette consumption OR smoking OR snus OR electronic cigarettes OR SU Smoking Cessation OR Tobacco Smoking OR Smokeless Tobacco OR TX smokeless OR “Smoking” OR “Tobacco” OR “Smoker” or “Smokers” OR Cigar* OR “Smokeless” OR E-cigarette* OR Electronic cigarette* OR “Snus” OR “Nicotine”OR "smoking-attributable deaths" OR "smoking attributable deaths" | Restriction: English and Human |
